# Characterization of the complete chloroplast genome sequence of *Maesa hupehensis* Rehd (primulaceae)

**DOI:** 10.1080/23802359.2022.2080023

**Published:** 2022-06-07

**Authors:** Yan Li, Xuan Yao, Qinxiang Chang, Caijuan Zhang, Pengguo Xia

**Affiliations:** a Department of Art Design, Taiyuan University, Taiyuan, China; b Key Laboratory of Plant Secondary Metabolism and Regulation of Zhejiang Province, College of Life Sciences and Medicine, Zhejiang Sci-Tech University, Hangzhou, China

**Keywords:** chloroplast genome, *Maesa hupehensis* Rehd, phylogenetic analysis

## Abstract

*Maesa hupehensis* Rehd 1916 is mainly planted in Hubei and Sichuan. In this study, we assembled and characterized the complete chloroplast genome of *M. hupehensis* as resources for the future study. The chloroplast genome was 157,005 bp in length, with 37.3% GC content, composing of one large single copy (87,628 bp) and one small single copy (18,111 bp), separated by two inverted repeats (25,633 bp). A total of 130 genes were predicted, including 8 rRNAs, 37 tRNAs, and 84 protein-coding genes. Phylogenetic analysis showed that *M. hupehensis* was closely related to *Maesa montana*.

The genus *Maesa* Forsk (Primulaceae) comprises ∼200 species mainly distributed in the tropics of the eastern hemisphere. There are 29 species and one variety in China, which are distributed in the south of the Yangtze River Basin (Lai et al. 2002). In previous classification of this genus, it was placed under the family Myrsinaceae. However, it belongs to the family Primulaceae based on gross analysis of morphological and gene data (APG III 2009; Wang and Xia 2011). *Maesa hupehensis* belongs to the genus *Maesa*, is a native plant of the Sichuan, Guangdong provinces in China. At present, the complete chloroplast information and systematic position of *M. hupehensis* has not been studied and reported. In this study, we sequenced, assembled and annotated the complete chloroplast genome of *M. hupehensis*, which will contribute to the further research of *Maesa* Forsk.

The materials of *M. hupehensis* came from Zhongba Canyon, Shiquan County, Ankang City, Shaanxi Province (32°54′56.11″N, 108°7′18.57″E, and altitude 527 m). A specimen was deposited at the Herbarium of Xi’an Botanical Garden (voucher number: *Xun Lulu* et al. *01495*, Lulu Xun, xunlulu20032006@126.com). The total genomic DNA of leaves was extracted by CTAB method (Doyle and Doyle 1987), sequenced using Illumina NovaSeq 6000 platform and filtered using Fastp software (Chen et al. 2018). The extracted DNA was deposited at Key Laboratory of Plant Secondary Metabolism and Regulation of Zhejiang Province, Zhejiang Sci-Tech University (http://sky.zstu.edu.cn) under the voucher number ZSTUX0101 (collected by Pengguo Xia and xpg_xpg@zstu.edu.cn). Resultant clean reads were assembled using GetOrganelle v1.7.0 (Jin et al. 2020). Geneious prime was used for gene annotation by having *Maesa montana* (KU569490) as the reference genome. Genomic data were deposited into NCBI GenBank with the accession number MZ846203.

The complete chloroplast genome of *M. hupehensis* was 157,005 bp in length containing two inverted repeat regions of 25,633 bp, a large single copy region of 87,628 bp and the small single copy region of 18,111 bp. The GC content was 37.3%. The complete chloroplast genome encoded 130 genes, including 84 protein coding genes, 37 tRNA genes, and 8 rRNA genes.

In order to determine the phylogenetic position of *M. hupehensis* in Primulaceae, the complete chloroplast genome sequences of 13 species from Primulaceae and two species of Ericaceae as complex outgroup were downloaded from GenBank, and all sequences were aligned using MAFFT (Katoh and Standley 2013). The best nucleotide substitution model was the Transversion model with empirical base frequencies and FreeRate model (TVM + F+R2), revealed by ModelFinder (Kalyaanamoorthy et al. 2017). The IQ-TREE v2.1.2 (Minh et al. 2020) was used for maximum likelihood (ML) reconstruction based on the selected model. Branch supports were calculated using ultrafast bootstrap (UFBoot) and SH-like approximate likelihood ratio test (SH-aLRT) with 1000 replicates (Figure 1). *Maesa hupehensis* was closely related to *M. montana* (KU569490). Our results provide valuable data and shed light on the phylogenomic study of Primulaceae.

**Figure 1.**
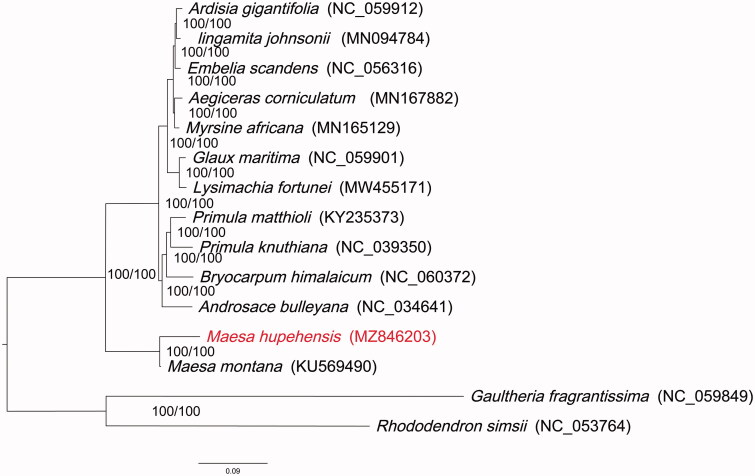
ML tree was constructed based on the complete chloroplast genome sequences of 13 species of *Primulaceae*, with *Gaultheria fragrantissima* and *Rhododendron simsii* of Ericaceae as outgroup species. *Maesa hupehensis* is indicated in red. Support values written on the branches: SH-aLRT support/ultrafast bootstrap support.

## Data Availability

The data that support the findings of this study are openly available in NCBI (https://www.ncbi.nlm.nih.gov) GenBank with the accession number (MZ846203). The associated BioProject, SRA, and BioSample numbers are PRJNA756405, SRR15533156, and SAMN20865416 respectively.
